# Association between Coronary Artery Measurements and Retinal Microvasculature in Children with New Onset of Kawasaki Disease

**DOI:** 10.1038/s41598-019-53220-3

**Published:** 2019-11-13

**Authors:** Edward Jianyang Lim, Izzuddin M. Aris, Jonathan Choo, Tien Yin Wong, Ling-Jun Li

**Affiliations:** 1 Yong Loo Lin School of Medicine, National University of Singapore, Singapore, Singapore; 2 Singapore Institute for Clinical Sciences, Agency for Science, Technology and Research, Singapore, Singapore; 3 Department of O&G, Yong Loo Lin School of Medicine, National University of Singapore, Singapore, Singapore; 4 Division of Chronic Disease Research Across the Lifecourse, Department of Population Medicine, Harvard Medical School and Harvard Pilgrim Health Care Institute, Boston, Massachusetts USA; 5 Cardiology Service, KK Women’s and Children’s Hospital, Singapore, Singapore; 6 Singapore Eye Research Institute, Singapore National Eye Centre, Singapore, Singapore; 7 OBGYN Academic Clinician Program (ACP), Duke-NUS Medical School, Singapore, Singapore; 8 Division of O&G, KK Women’s and Children’s Hospital, Singapore, Singapore

**Keywords:** Cardiology, Congenital heart defects, Eye diseases

## Abstract

About a quarter of children with new onset of Kawasaki disease (KD) encounter coronary arterial involvement. While KD is known to cause vasculitis of medium-sized vessels, few studies have been done to study the involvement of the microcirculation. We aimed to investigate the association between coronary arterial dilatation and retinal microvasculature in a pilot setting, in order to further study the pathophysiological mechanism of KD from the perspective of small vessels changes. We performed a cross-sectional, observational, hospital-based study on 11 children aged 2 years and above with new-onset KD. Cardiac imaging technicians performed the echocardiographic examinations and recorded right coronary artery (RCA), left coronary artery (LCA) and left anterior descending artery (LAD). Qualified retinal graders reviewed and graded standardised retinal photographs to assess retinal microvascular parameters. Among 11 participants, there were 7 boys and 4 girls. Median and interquartile range of participants’ age were 5.92 (3.08) years. After adjusting for age and sex, each unit increase in LAD (mm) was significantly associated with increment of retinal arteriolar tortuosity (4.25 × 10^−5^ units, 95% Confidence Interval: 1.19, 7.32). Retinal arteriolar geometric changes were associated with LAD dilatation in 11 children with new onset of KD. Our pilot provided proof-of-concept that retinal imaging might be useful for detecting coronary arterial involvement in young children with KD and it needs further investigation.

## Introduction

Kawasaki Disease (KD) is a childhood vasculitis that has become increasingly common in developed countries^1^. Although the acute inflammatory phase is self-limited, cardiac complications may develop and these cause significant long term morbidity and mortality in 20–25% of untreated paediatric patients^2^. Early diagnosis and treatment is crucial for KD patients, in order to prevent mortality that peaks 15 to 45 days after the initial onset of fever^3^. The pathophysiology of KD arteriopathy has been widely speculated to be characterised by 3 linked processes of necrotising arteritis, subacute/chronic vasculitis and luminal myofibroblastic proliferation. While KD is known to cause vasculitis of medium-sized vessels, few studies have been done to study the involvement of the microcirculation. Therefore, there is a need to further study pathophysiological mechanism of KD from the perspective of small vessel changes. As such, examining retinal vasculature in a non-invasive manner may enhance our ability to explore KD pathogenesis via microcirculation among paediatric patients with new-onset KD.

Retinal imaging technology has enabled researchers to assess changes in retinal microvascular morphology (e.g. vascular calibre, curvature tortuosity, fractal dimension and branching angle) in a non-invasive and accurate manner. Such systematic parameters mirror an “optimal state” of the retinal microcirculation, which may also reflect systemic microcirculation^4^. A previous cross-sectional study conducted on Singaporean and Australian chronic paediatric KD patients, who suffered relapses and were treated for years, found that wider retinal venules were associated with coronary artery abnormalities, such as giant coronary artery aneurysms^5^. However, it is still unknown whether such retinal vascular changes exist during the new-onset other than recurrent acute phase of KD. If such proof-of-concept is proven, it is worth exploring the potential screening value of retinal technology for coronary arterial dilation detection among young children with new-onset KD, in addition to conventional screening techniques.

In this pilot study, we examined the association of cardiac measures and retinal microvasculature in 11 young children with new-onset KD. We hypothesised that early dilatation of coronary arteries is associated with a series of changes in retinal microvasculature (e.g. retinal arteriolar dilation), and it would be worth exploring further in terms of the screening value of retinal imaging among children at risk of coronary arterial involvement with new-onset KD.

## Methods

In order to minimise the possibility of unintentionally sharing information that can be used to re-identify private information, the data and study materials that support the findings of this study will not be available. This is a cross-sectional, observational, hospital-based study on new-onset of paediatric KD patients. We recruited patients during their first ward admission before intravenous immunoglobulin (IVIG) treatment was given, at the KK Women’s and Children’s Hospital, Singapore. We considered all children eligible for the study if they were: (1) aged 2 years and above; (2) had newly diagnosed and untreated KD; and (3) without other comorbidities during the period of assessment. We excluded patients who were suffering from any active eye diseases (e.g. keratitis) except for refractive error. We screened 14 patients from May 2015 to July 2016. A total of 11 patients (median 5.92 years, inter-quartile range 3.08 years) were included for final analysis (response rate 78.6%) after excluding three patients [2 declined participation and 1 with ungradable retinal photos]. We conducted the study according to the tenets of the Declaration of Helsinki, and obtained approval from the SingHealth Centralized Institutional Review Board and the National Health Group’s Domain Specific Review Board. Trained research coordinators obtained written informed consents from the legal guardians of all paediatric patients.

Cardiac imaging technicians performed the echocardiographic examinations and recorded cardiac measures such as right coronary artery (RCA), left coronary artery (LCA) and left anterior descending artery (LAD). Cardiologists reviewed all scans to ensure the accuracy of the cardiac measures. We calculated z-scores of RCA, LCA and LAD based on the recently published guidelines in a large series of US children with all races and ethnicities (n = 3566), using the equation z = [(x/BSA^α^) − μ]/σ, where x is the observed parameter value, BSA is the body surface area, α is the exponential power to be used with BSA, μ is the mean and σ is the standard deviation^6^. The BSA measurements were derived using the Haycock Formula, BSA (m^2^) = 0.024265 × height (cm)^0.3964^ × weight (kg)^0.5378^ ^7^. We also collected systolic blood pressure (SBP) and anthropometric measures to calculate body mass index (BMI). The SBP z-scores were calculated based on the very large database from fourth report from the National High Blood Pressure Education Program Working Group on Children and Adolescents (n = 63,227), using the equation z = (x − μ)/σ, where x is the observed parameter value, μ is the mean and σ is a given value from the appendix table in the report^8^. The BMI z-scores were calculated based on the United States Growth Charts, National Center for Health Statistics, using the equation z = [(x/M)**L − 1]/LS, L ≠ 0 or z = ln (x/M)/S, L = 0, where x is the observed parameter value, L, M and S are the values from the appropriate table corresponding to the age in months of the child and (x/M)**L is raising the quantity (X/M) to the Lth power^9^.

We took retinal photographs within the same day of definite KD diagnosis and yet before IVIG administration. However, if the KD diagnosis had been unclear via ECHO exam for the first time and the patient was temporarily diagnosed as incomplete KD (defined as children are suspected of having KD who do not fulfil diagnostic criteria), IVIG would still be given by the paediatric cardiologists, in order to prevent the patients from developing other manifestations subsequently. And therefore, we performed retinal photography 1–2 days after a re-examination on ECHO with a definite diagnosis and yet after IVIG treatment. Of the 11 subjects, 5 subjects received IVIG treatment before retinal photographs were taken. We used a non-mydriatic retinal camera (CR-DGi, Canon, Tokyo, Japan) to obtain one optic nerve centre and one macula centre photo for each eye, without any pupil dilation. Qualified retinal graders reviewed and graded retinal photographs using a semi-automated software (Singapore I Vessel Analyser [SIVA], version 4.0, Singapore Eye Research Institute, Singapore), which assessed retinal microvascular parameters including calibre, branching angle, fractal dimension and tortuosity, according to standardized protocols^10^ (Supplementary Figure 1). We assessed intra-grader reliability using 10% (n = 2) of randomly selected retinal photographs from our study, and the intra-class correlation coefficient was above 0.80 for all retinal vascular measures.

We examined the association between coronary arterial measures and retinal vascular measures using linear regression in two models: Model 1, unadjusted and Model 2, adjusted for child age and sex. We used SPSS 19.0 (IBM Analytics, Chicago, US) to conduct all statistical analyses. A 95% confidence interval (CI) and a 5% level of significance were utilised; therefore, we took statistical significance for the p value to be less than 0.05 for the two-tailed test.

## Results

Among 11 patients, majority were males (63.6%) and of Chinese ethnicity (81.8%). There were no sex differences in all of the measures. Table 1 shows other characteristics of the patients.

**Table 1:** Characteristics of 11 KD paediatric patients in our study.

Pre-IVIG Characteristics	All (n = 11)	Male (n = 7)	Female (n = 4)
	Median (IQR) or n (%)	Median (IQR) or n (%)	Median (IQR) or n (%)
Age	5.92 (3.08)	6.17 (3.08)	4.46 (2.58)
Sex			
Male	7 (63.6%)	—	—
Race/Ethnicity			
Chinese	9 (81.8%)	6 (54.5%)	3 (27.3%)
Malay	1 (9.1%)	1 (9.1%)	0 (0%)
Caucasian	1 (9.1%)	0 (0%)	1 (9.1%)
Blood Pressure and Anthropometry			
SBP, mmHg	98.0 (23.0)	99.0 (31.0)	94.0 (21.5)
SBP z-score	1.03 (2.10)	1.15 (2.88)	0.51 (1.80)
BMI, kg/m^2^	14.7 (2.6)	13.9 (2.7)	15.8 (2.1)
BMI z-score	−0.46 (2.91)	−1.38 (2.67)	0.21 (1.46)
BSA, m^2^	0.69 (0.20)	0.73 (0.15)	0.64 (0.14)
Laboratory Markers			
ESR, mm/h	96.0 (34.0)	74.0 (37.0)	99.5 (15.3)
CRP, mg/L	68.5 (94.8)	68.5 (54.7)	77.2 (153.8)
WBC, x10^9^/L	14.7 (5.2)	15.5 (10.5)	12.0 (2.9)
Hb, g/dL	11.0 (1.6)	11.2 (1.3)	10.6 (1.9)
Echo Measures			
RCA, mm	1.77 (0.41)	1.77 (0.36)	1.77 (0.73)
RCA z-score	−0.30 (1.06)	−0.30 (1.06)	−0.32 (1.25)
LCA, mm	2.26 (0.37)	2.26 (0.29)	2.35 (0.40)
LCA z-score	−0.48 (0.89)	−0.69 (1.27)	−0.06 (0.44)
LAD^|^, mm	1.78 (0.32)	1.82 (0.25)	1.62 (0.48)
LAD z-score	0.43 (0.78)	0.43 (0.62)	0.24 (1.32)
Retinal Vessels Parameters			
Arteriolar diameter, µm	127.62 (9.60)	127.62 (9.60)	126.76 (23.00)
Venular diameter, µm	182.47 (15.01)	179.24 (17.41)	187.04 (12.28)
Arteriolar fractal dimension, Df	1.22 (0.067)	1.22 (0.067)	1.22 (0.12)
Venular fractal dimension, Df	1.21 (0.050)	1.21 (0.053)	1.20 (0.13)
Arteriolar curvature tortuosity, unit (^−5^)	13.86 (5.20)	15.26 (5.89)	10.70 (3.58)
Venular curvature tortuosity, unit (^−5^)	12.44 (2.22)	11.90 (2.93)	12.64 (3.42)
Arteriolar branching angle, degree	86.93 (10.75)	90.93 (16.92)	85.58 (7.88)
Venular branching angle, degree	82.95 (19.99)	82.28 (16.52)	86.01 (17.84)

Abbreviations: IVIG, Intravenous immunoglobulin; SBP, Systolic blood pressure; BMI, Body mass index; BSA, Body surface area; ESR, Erythrocyte sedimentation rate; CRP, C-reactive protein; WBC, White blood cell; Hb, Haemoglobin; RCA, Right coronary artery; LCA, Left coronary artery; LAD, Left anterior descending artery.

Tables 2 and 3 show the associations between coronary artery parameters and retinal vessel parameters in patients with new-onset KD. In the unadjusted model, each 1.0 mm increase in RCA was significantly associated with increased retinal arteriolar fractal dimension (0.11 Df; 95% CI: 0.33 × 10^−3^, 0.22). Each 1.0 mm increase in LAD was significantly associated with increment of retinal arteriolar branching angle (11.46 degrees; 0.77, 22.16) and retinal arteriolar curvature tortuosity (4.78 × 10^−5^ units; 2.30, 7.27) (Table 2). Furthermore, each unit increase in LAD z-score was significantly associated with increased retinal arteriolar fractal dimension (0.06 Df; 0.39 × 10^−3^, 0.11) (Table 3). However, after adjusting for age and sex, only the association between LAD and retinal arteriolar tortuosity remained significant (4.25 × 10^−5^ units, 95% CI: 1.19, 7.32) (Table 2). We performed a sensitivity analysis additionally adjusting for IVIG treatment (yes vs. no), which did not change our estimates significantly.

**Table 2:** Association between coronary artery parameters and retinal vessel parameters in patients with new onset of Kawasaki disease.

Retinal microvascular measures		Echo Measures		
		RCA per 1 mm ↑	LCA per 1 mm ↑	LAD per 1 mm ↑
Retinal Arteriolar Diameter, µm				
Model 1	β, 95% CI P value	1.61 (−24.20, 27.41) 0.89	−4.81 (−22.88, 13.27) 0.56	−0.36 (−15.43, 14.71) 0.96
Model 2	β, 95% CI P value	2.60 (−30.22, 35.43) 0.86	−4.92 (−26.23, 16.39) 0.60	0.86 (−20.02, 21.74) 0.93
Retinal Venular Diameter, µm				
Model 1	β, 95% CI P value	−10.27 (−41.16, 20.63) 0.47	−8.61 (−30.39, 13.17) 0.40	2.46 (−16.03, 20.95) 0.77
Model 2	β, 95% CI P value	−10.34 (−50.00, 29.32) 0.56	−8.41 (−34.27, 17.45) 0.47	2.08 (−23.71, 27.86) 0.85
Retinal Arteriolar Fractal Dimension, Df				
Model 1	β, 95% CI P value	***0.11 (0.33*** **×** ***10***^***−3***^***, 0.22)*** ***0.05***	−0.30 × 10^−2^ (−0.10, 0.09) 0.95	0.03 (−0.05, 0.11) 0.39
Model 2	β, 95% CI P value	0.11 (−0.03, 0.25) 0.09	−0.40 × 10^−2^ (−0.12, 0.11) 0.94	0.04 (−0.06, 0.15) 0.35
Retinal Venular Fractal Dimension, Df				
Model 1	β, 95% CI P value	0.05 (−0.08, 0.18) 0.38	−0.33 (−0.12, 0.06) 0.42	−0.02 (−0.09, 0.06) 0.59
Model 2	β, 95% CI P value	0.03 (−0.12, 0.19) 0.64	−0.04 (−0.13, 0.06) 0.41	−0.02 (−0.12, 0.07) 0.57
Retinal Arteriolar Branching Angle, degree				
Model 1	β, 95% CI P value	2.73 (−20.74, 26.21) 0.80	3.71 (−12.88, 20.29) 0.63	***11.46 (0.77, 22.16)*** ***0.04***
Model 2	β, 95% CI P value	8.17 (−18.05, 34.39) 0.49	4.66 (−12.86, 22.17) 0.55	10.79 (−3.55, 25.13) 0.12
Retinal Venular Branching Angle, degree				
Model 1	β, 95% CI P value	1.17 (−26.71, 29.05) 0.93	−4.15 (−23.81, 15.51) 0.64	3.05 (−13.06, 19.16) 0.68
Model 2	β, 95% CI P value	2.89 (−31.71, 37.49) 0.85	−4.33 (−26.94, 18.29) 0.67	7.05 (−14.06, 28.15) 0.46
Retinal Arteriolar Curvature Tortuosity, unit ^–5^				
Model 1	β, 95% CI P value	1.01 (−6.45, 8.48) 0.77	1.84 (−3.33, 7.02) 0.44	***4.78 (2.30, 7.27)*** ***0.002***
Model 2	β, 95% CI P value	1.31 (−6.29, 8.91) 0.70	2.10 (−2.63, 6.83) 0.33	***4.25 (1.19, 7.32)*** ***0.01***
Retinal Venular Curvature Tortuosity, unit ^–5^				
Model 1	β, 95% CI P value	−0.08 (−5.47, 5.32) 0.98	0.51 (−3.33, 4.34) 0.77	1.92 (−0.88, 4.72) 0.16
Model 2	β, 95% CI P value	1.73 (−3.49, 6.94) 0.46	0.80 (−2.72, 4.33) 0.61	1.22 (−2.06, 4.49) 0.41

Abbreviations: RCA, Right coronary artery; LCA, Left coronary artery; LAD, Left anterior descending artery; CI, Confidence interval.

^|^Model 1, unadjusted;

^#^Model 2, adjusted for age and sex.

**Table 3:** Association between coronary artery parameters z-scores and retinal vessel parameters in patients with new onset of Kawasaki disease.

Retinal microvascular measures		Echo measures			
		RCA, z-score Per unit ↑	LCA, z-score Per unit ↑		LAD, z-score Per unit ↑
**Retinal Arteriolar Diameter, µm**					
Model 1	β, 95% CI P value	3.21 (−9.96, 16.38) 0.60	2.30 (−8.21, 12.80) 0.63	3.18 (−10.15, 16.51) 0.60	
Model 2	β, 95% CI P value	3.68 (−11.93, 19.29) 0.60	2.42 (−11.11, 15.96) 0.69	3.59 (−12.83, 20.02) 0.62	
**Retinal Venular Diameter, µm**					
Model 1	β, 95% CI P value	−1.53 (−18.00, 14.94) 0.84	4.93 (−7.65, 17.51) 0.40	0.20 (−16.49, 16.90) 0.98	
Model 2	β, 95% CI P value	−1.56 (−21.25, 18.13) 0.86	5.16 (−11.17, 21.48) 0.48	0.97 (−19.73, 21.66) 0.92	
**Retinal Arteriolar Fractal Dimension, Df**					
Model 1	β, 95% CI P value	0.05 (−0.01, 0.11) 0.09	−0.40 × 10^−2^ (−0.06, 0.05) 0.86	***0.06 (0.39*** **×** ***10***^***−3***^***, 0.11)*** ***0.05***	
Model 2	β, 95% CI P value	0.05 (−0.02, 0.12) 0.15	0.12 × 10^−2^ (−0.70, 0.07) 0.97	0.06 (−0.01, 0.13) 0.09	
**Retinal Venular Fractal Dimension, Df**					
Model 1	β, 95% CI P value	0.02 (−0.04, 0.09) 0.44	−0.42 × 10^−2^ (−0.06, 0.05) 0.87	0.02 (−0.05, 0.08) 0.61	
Model 2	β, 95% CI P value	0.02 (−0.05, 0.09) 0.54	0.76 × 10^−2^ (−0.06, 0.07) 0.79	0.80 × 10^−2^ (−0.07, 0.09) 0.82	
**Retinal Arteriolar Branching Angle, degree**					
Model 1	β, 95% CI P value	4.22 (−7.58, 16.01) 0.44	6.53 (−1.84, 14.90) 0.11	3.09 (−9.04, 15.22) 0.58	
Model 2	β, 95% CI P value	4.11 (−8.56, 16.78) 0.47	5.66 (−4.49, 15.80) 0.23	5.70 (−7.17, 18.57) 0.33	
**Retinal Venular Branching Angle, degree**					
Model 1	β, 95% CI P value	0.77 (−13.68, 15.22) 0.91	0.37 (−11.11, 11.86) 0.94	−2.89 (−17.35, 11.57) 0.66	
Model 2	β, 95% CI P value	1.59 (−15.17, 18.34) 0.83	−0.26 (−14.71, 14.19) 0.97	−3.03 (−20.47, 14.41) 0.69	
**Retinal Arteriolar Curvature Tortuosity, unit** ^**–5**^					
Model 1	β, 95% CI P value	0.83 (−3.01, 4.67) 0.64	1.51 (−1.37, 4.38) 0.27	1.20 (−2.63, 5.03) 0.50	
Model 2	β, 95% CI P value	0.40 (−3.31, 4.11) 0.81	1.78 (−1.00, 4.56) 0.18	1.66 (−1.96, 5.28) 0.32	
**Retinal Venular Curvature Tortuosity, unit** ^**–5**^					
Model 1	β, 95% CI P value	0.42 (−2.36, 3.20) 0.74	0.55 (−1.63, 2.74) 0.58	−1.28 (−3.94, 1.38) 0.30	
Model 2	β, 95% CI P value	0.44 (−2.17, 3.05) 0.70	−0.14 (−2.40, 2.12) 0.89	−0.63 (−3.34, 2.08) 0.60	

Abbreviations: RCA, Right coronary artery; LCA, Left coronary artery; LAD, Left anterior descending artery; CI, Confidence interval.

^|^Model 1, unadjusted;

^#^Model 2, adjusted for age and sex.

## Discussion

In this hospital-based, cross-sectional study among children with new-onset of KD, a larger left anterior descending artery index was associated with greater values in retinal arteriolar geometric parameters especially curvature tortuosity.

A previous study found that increment of coronary artery diameters was associated with wider retinal venules in paediatric KD patients with recurrent acute flares^5^. We found however, that retinal arteriolar changes, instead of retinal venular changes, were associated with coronary artery changes during the acute phase of new KD onset. We speculated that the retinal venular changes in the published study might be a result of general upregulated systemic inflammation^1^, instead of specific features of retinal vascular morphology related to KD. In our study, we reported a series of changes in retinal arteriolar geometric features including greater fractal dimension and curvature tortuosity. As suggested in previous studies, fractal dimension measures the degree of geometric complexity^11^, while tortuosity assesses the shape of retinal vessel^12^. Both geometric parameters reflect an optimal blood circulation in the retina. Thus, altered fractal dimension and/or curvature tortuosity could reflect increased angiogenesis or oxidative stress in response to inflammatory-induced neuro-retinal hypoxia and endothelial dysfunction^5^. Although increased retinal tortuosity has been speculated to be related to increased blood flow and angiogenesis^13–15^, the true physiologic significance of increased retinal arteriolar tortuosity is still not fully understood. Aside from inflammation and systemic vascular conditions, other causes of increased retinal arteriolar tortuosity include established cardiovascular risk factors including increased age, BMI, BP triglyceride levels, total and low-density lipoprotein cholesterol^12,16^. Due to inflammatory-mediated mechanisms in KD coronary vasculopathy^2^, retinal arteriolar changes in our study could reflect the degree of KD related inflammation and might even indicate the degree of coronary arterial damage. Estimates of all associations in our study were either attenuated or diminished after the application of coronary artery z-scores, which were calculated using BSA. As previous studies have reported strong associations between BMI and/or BSA with retinal vascular calibre among the paediatric population^17,18^, we speculated a possible BSA effect on the observed relationships between coronary artery structure and retinal microvasculature.

The strength of our study includes the use of quantitative measurements of both cardiac and retinal vascular measures, which is novel in a group of young children with new-onset of KD and the adoption of ECHO measures z-scores based on a large multi-race/ethnicity paediatric population. However, there are some potential limitations. First, the cross-sectional nature of the study design limits our interpretation on the temporal relationship of coronary artery parameters and retinal vascular parameters. Second, our findings may be biased by selection as patients were recruited only from a single paediatric hospital in Singapore, which may not be generalisable to other paediatric KD patients. Third, the small sample size limits the statistical power to detect other associations of retinal microvascular and cardiac parameters. Fourth, the adoption of z-scores referencing mainly American paediatric population instead of Asian paediatric subjects may have biased our observations. Lastly, our study did not correct for multiple comparisons and adjust for more confounders due to a small sample size.

Our study provided the proof-of-concept of significant associations between coronary arterial dilation and retinal arteriolar changes in a pilot setting. The retinal photography process was fast and non-invasive. For paediatric patients between 3–4 years old, the retinal photography, on average, took 15 minutes. For older or more cooperative paediatric patients, this process was shortened to <10 minutes. Future studies with larger samples and longer follow-up periods are warranted to further explore the potential screening values of retinal microvasculature in children, in terms of detecting coronary arterial involvements in the acute phase of new-onset KD. Further studies are also warranted to obtain post-treatment changes in ECHO and retinal parameters in order to determine the duration and direction of both changes in coronary and retinal vessels. Additionally, future studies should explore whether the magnitude of vessel changes are more pronounced among children with KD who have abnormal z-scores.

## Supplementary information

Retinal microvasculature assessment on the grading platform.

## Data Availability

The datasets generated during and/or analysed during the current study are not publicly available to minimise the possibility of unintentionally sharing information that can be used to re-identify private information, but are available from the corresponding author on reasonable request.
