# Supplementary material for: Association between Coronary Artery Measurements and Retinal Microvasculature in Children with New Onset of Kawasaki Disease
Source: Sci Rep. 2019 Nov 13;9:16714. doi: 10.1038/s41598-019-53220-3 (PMC6853953; doi:10.1038/s41598-019-53220-3)
Supplement: Supplementary file 1 — Retinal microvasculature assessment on the grading platform. [file 41598_2019_53220_MOESM1_ESM.pdf]

Association between Coronary Artery Measurements and Retinal Microvasculature in Children with New Onset of Kawasaki Disease

Edward Jianyang Lim,[1] Izzuddin M. Aris PhD,[2,3,4,] Jonathan Choo MD,[5] Tien Yin Wong,[6] Ling-Jun Li MD, PhD[6,7,8*]

[1] Yong Loo Lin School of Medicine, National University of Singapore, Singapore

[2] Singapore Institute for Clinical Sciences, Agency for Science, Technology and Research, Singapore, Singapore

[3] Department of O&G, Yong Loo Lin School of Medicine, National University of Singapore, Singapore

[4] Division of Chronic Disease Research Across the Lifecourse, Department of Population Medicine, Harvard Medical School and Harvard Pilgrim Health Care Institute, Boston, Massachusetts, USA

[5] Cardiology Service, KK Women's and Children's Hospital, Singapore

[6] Singapore Eye Research Institute, Singapore National Eye Centre, Singapore

[7] OBGYN Academic Clinician Program (ACP), Duke-NUS Medical School, Singapore

[8] Division of O&G, KK Women's and Children's Hospital, Singapore

*Corresponding author: Ling-Jun Li, M.D., Ph.D, Division of OBGYN, KK Women's and Children's Hospital, 100 Bukit Timah Road, Singapore 229899. (DID): +65 6394 1099; (FAX): +65 6394 2241. Email: queenie.li.l@kkh.com.sg.

Supplementary Figure 1. Retinal microvasculature assessment on the grading platform.

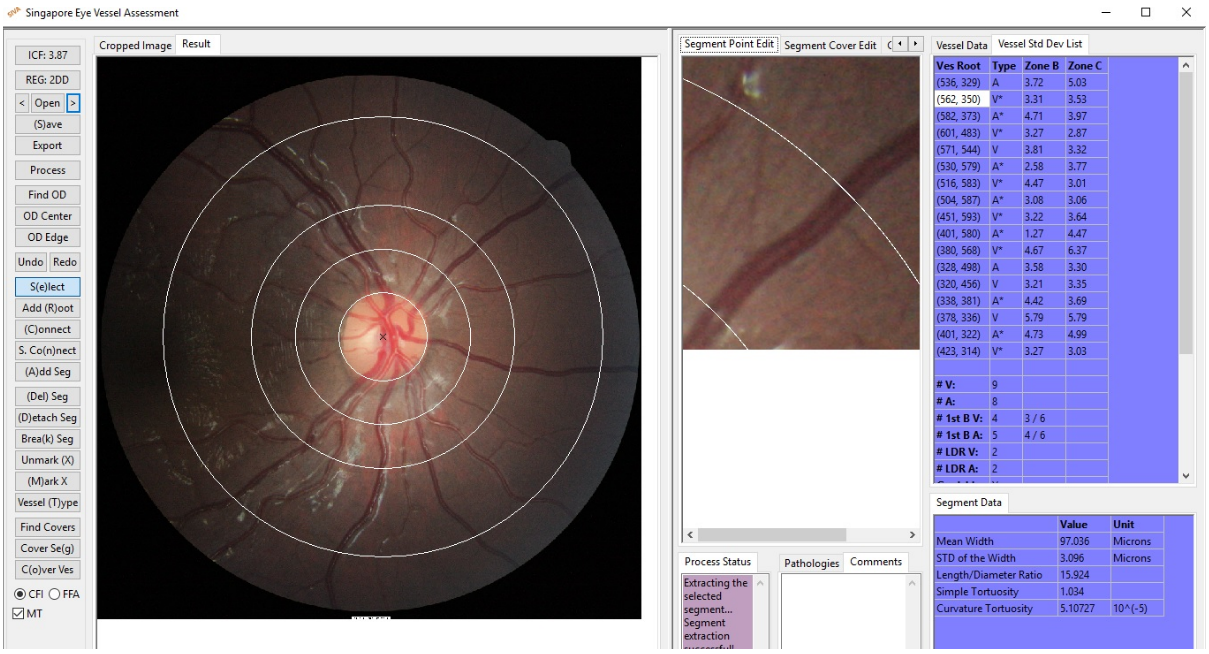

Left: Retinal fundus photograph with automated zone demarcation by the computer-assisted programme (SIVA). Zone C is demarcated in SIVA by 0.5 to 2.0 optic disc diameters away from the margin of the optic disc; *Right:* Arterioles and venules larger than 25 μm are assessed within zone C.
